# Saccharomyces cerevisiae Gene Expression during Fermentation of Pinot Noir Wines at an Industrially Relevant Scale

**DOI:** 10.1128/AEM.00036-21

**Published:** 2021-05-11

**Authors:** Taylor Reiter, Rachel Montpetit, Shelby Byer, Isadora Frias, Esmeralda Leon, Robert Viano, Michael Mcloughlin, Thomas Halligan, Desmon Hernandez, Ron Runnebaum, Ben Montpetit

**Affiliations:** a Food Science Graduate Group, University of California, Davis, Davis, California, USA; b Department of Viticulture and Enology, University of California, Davis, Davis, California, USA; c Department of Population Health and Reproduction, University of California, Davis, Davis, California, USA; d Department of Chemical Engineering, University of California, Davis, Davis, California, USA; The Pennsylvania State University

**Keywords:** *Saccharomyces cerevisiae*, fermentation, gene expression

## Abstract

Saccharomyces cerevisiae metabolism produces ethanol and other compounds during the fermentation of grape must into wine. Thousands of genes change expression over the course of a wine fermentation, allowing S. cerevisiae to adapt to and dominate the fermentation environment. Investigations into these gene expression patterns previously revealed genes that underlie cellular adaptation to the grape must and wine environments, involving metabolic specialization and ethanol tolerance. However, the majority of studies detailing gene expression patterns have occurred in controlled environments that may not recapitulate the biological and chemical complexity of fermentations performed at production scale. Here, an analysis of the S. cerevisiae RC212 gene expression program is presented, drawing from 40 pilot-scale fermentations (150 liters) using Pinot noir grapes from 10 California vineyards across two vintages. A core gene expression program was observed across all fermentations irrespective of vintage, similar to that of laboratory fermentations, in addition to novel gene expression patterns likely related to the presence of non-*Saccharomyces* microorganisms and oxygen availability during fermentation. These gene expression patterns, both common and diverse, provide insight into Saccharomyces cerevisiae biology critical to fermentation outcomes under industry-relevant conditions.

**IMPORTANCE** This study characterized Saccharomyces cerevisiae RC212 gene expression during Pinot noir fermentation at pilot scale (150 liters) using industry-relevant conditions. The reported gene expression patterns of RC212 are generally similar to those observed under laboratory fermentation conditions but also contain gene expression signatures related to yeast-environment interactions found in a production setting (e.g., the presence of non-*Saccharomyces* microorganisms). Key genes and pathways highlighted by this work remain undercharacterized, indicating the need for further research to understand the roles of these genes and their impact on industrial wine fermentation outcomes.

## Abstract (precis)

This study characterized Saccharomyces cerevisiae RC212 gene expression during Pinot noir fermentation at pilot scale (150 liters) using industry-relevant conditions. The reported gene expression patterns of RC212 are generally similar to those observed under laboratory fermentation conditions but also contain gene expression signatures related to yeast-environment interactions found in a production setting (e.g., the presence of non-*Saccharomyces* microorganisms).

## INTRODUCTION

Saccharomyces cerevisiae is most often the dominant fermentative organism during vinification. As a domesticated species, it has evolved specialized metabolic strategies to assimilate sugars in grape must and to transform them into ethanol, thereby outcompeting other microorganisms during fermentation (1). During this process, S. cerevisiae encounters a dynamic stress landscape. In early fermentation, sources of stress include high sugar concentrations (osmotic stress), low pH (acid stress), decreasing oxygen (hypoxia), the presence of other organisms that compete for nutrients or produce inhibitory compounds, and sulfur dioxide additions that are used to inhibit spoilage organisms. As fermentation progresses, nutrients become limiting (starvation), the temperature may rise or be kept low (heat/cold stress), and ethanol concentrations rise (ethanol stress). However, through a coordinated gene expression response, S. cerevisiae adapts to these stresses and most often continues fermentation until the must contains no residual sugar.

High-throughput gene expression profiling (e.g., microarray and high-throughput RNA sequencing [RNA-seq]) has offered a window into the metabolic strategies used by S. cerevisiae during fermentation to adapt to and to dominate fermentation environments. Previous research reported expression changes in >2,000 genes during fermentation (2–4). In early fermentation, this is marked by expression of gene products that support biosynthetic processes and acquisition of abundant nutrient resources (2, 3). As fermentation progresses, nitrogen limitation, phosphate limitation, and/or ethanol accumulation can trigger a transition to a nonproliferative state (i.e., stationary phase), which involves remodeling the gene expression program to support cellular adaptation to the changing environment with continued metabolism (2, 3). Toward the end of fermentation, relief of nitrogen catabolite repression (2) and increased expression of nitrogen recycling genes (2, 5) is observed, which can be accompanied by further remodeling of the translational machinery and increased oxidative metabolism (5, 6). As ethanol concentrations rise through the end of fermentation, a gradual transcriptome response to ethanol stress is also observed (3). This response overlaps, but appears distinct from, the environmental stress response (ESR) seen in laboratory yeast (2, 3, 7), which may be related to the multitude of simultaneous stresses experienced by the yeast at the end of wine fermentation. Indeed, the majority of genes with stress response elements in their promoters are expressed at the end of fermentation (8).

Through the associated metabolic processes that consume and produce a multitude of compounds, S. cerevisiae gene expression in response to environmental factors is related to overall fermentation kinetics and wine sensory outcomes. For example, fermentations can become sluggish or stuck when S. cerevisiae inadequately adapts to stresses encountered in the wine fermentation environment (9). In addition, altered gene expression likely underlies different wine sensory characteristics in fermentations conducted with different industrial yeast strains (10, 11). To impact wine quality, genetic strategies have been applied in attempts to alter the expression of flavor-associated genes (12), which have achieved variable levels of success. Consequently, further study of the S. cerevisiae gene expression program across fermentation is required to understand the yeast-environment relationship and how these interactions may be controlled to alter fermentation outcomes.

Given the importance of the yeast-environment interaction in determining gene expression, a major consideration with respect to collecting such data is the fermentation conditions used. To date, the majority of gene expression surveys have profiled fermentations that deviate in one or more ways from the industrial conditions in which most fermentations take place. For example, hundreds to thousands of liters of grape must are fermented to wine at industrial scales, while milliliter to liter volumes are commonly used in laboratory studies of gene expression (2–5, 13–16). Industry-scale fermentations also have different kinetics, compared with laboratory-scale fermentations (4, 15, 17), and are less aromatic due to differences in hydrodynamics (15, 18). Similarly, dissolved oxygen levels differ at laboratory scale, compared with industry scale (4), which can impact fermentation outcomes (19, 20). Possibly reflecting these different environments, at the end of fermentation the expression of key genes involved in amino acid transport and other core metabolic processes have been shown to differ between laboratory and industrial fermentations (4). Consequently, we propose that the physical and chemical differences in laboratory-scale versus industry-scale wine fermentations are important factors to consider when analyzing gene expression patterns across fermentation.

Another major consideration when conducting gene expression studies is that most studies investigate the fermentative capability of S. cerevisiae in monoculture using sterile synthetic media or filter-sterilized grape must (2–5, 12, 21). These controlled studies are important and allow connections between the media, gene expression, and wine outcomes to be made (12) but do not recapitulate the complexity of a natural grape must that varies in parameters such as nitrogen composition, pH, and phenolic and elemental profiles (22–25). In addition, these experiments lack the diverse grape must microbiome that is a contributing component of wine fermentations (26–40). These are all parameters that shape the fermentation environment and the metabolic response of S. cerevisiae.

Interspecies interactions are a critical component of the fermentation environment that informs the biology and behavior of S. cerevisiae during fermentation. It has been shown that non-*Saccharomyces* yeast impact the metabolism of S. cerevisiae through direct and indirect interactions (41–43), leading to faster resource acquisition by S. cerevisiae in early fermentation and altered metabolism of vitamins and minerals (42–45). While research is still needed to describe the impact of a diverse microbial consortia on S. cerevisiae during fermentation (46, 47), it remains that industrial fermentations are not sterile and involve diverse microorganisms (30, 36, 37, 39, 40). Even in fermentations treated with sulfur dioxide (SO_2_) to control microbial spoilage organisms, native fungi and bacteria are metabolically active during fermentation (40, 48, 49). This makes profiling S. cerevisiae gene expression among diverse microbial consortia important, as it will lead to a better understanding of the principles that govern S. cerevisiae gene expression and metabolism during fermentation.

Here, to begin to address the impact of an industrial wine fermentation environment on S. cerevisiae gene expression, the inherent variability found in industrial fermentations was incorporated to determine the S. cerevisiae RC212 gene expression program across chemically and biologically diverse Pinot noir grape musts. Specifically, time-series RNA-seq was used to capture the gene expression profiles of RC212 during 40 inoculated primary fermentations at pilot scale (150 liters) using California Pinot noir grapes from 10 vineyards across two vintages. Using differential expression across the continuous variable Brix, the core gene expression program used by S. cerevisiae during these fermentations was observed.

## RESULTS AND DISCUSSION

### Conditions and rates of fermentation.

**FIG 1:**
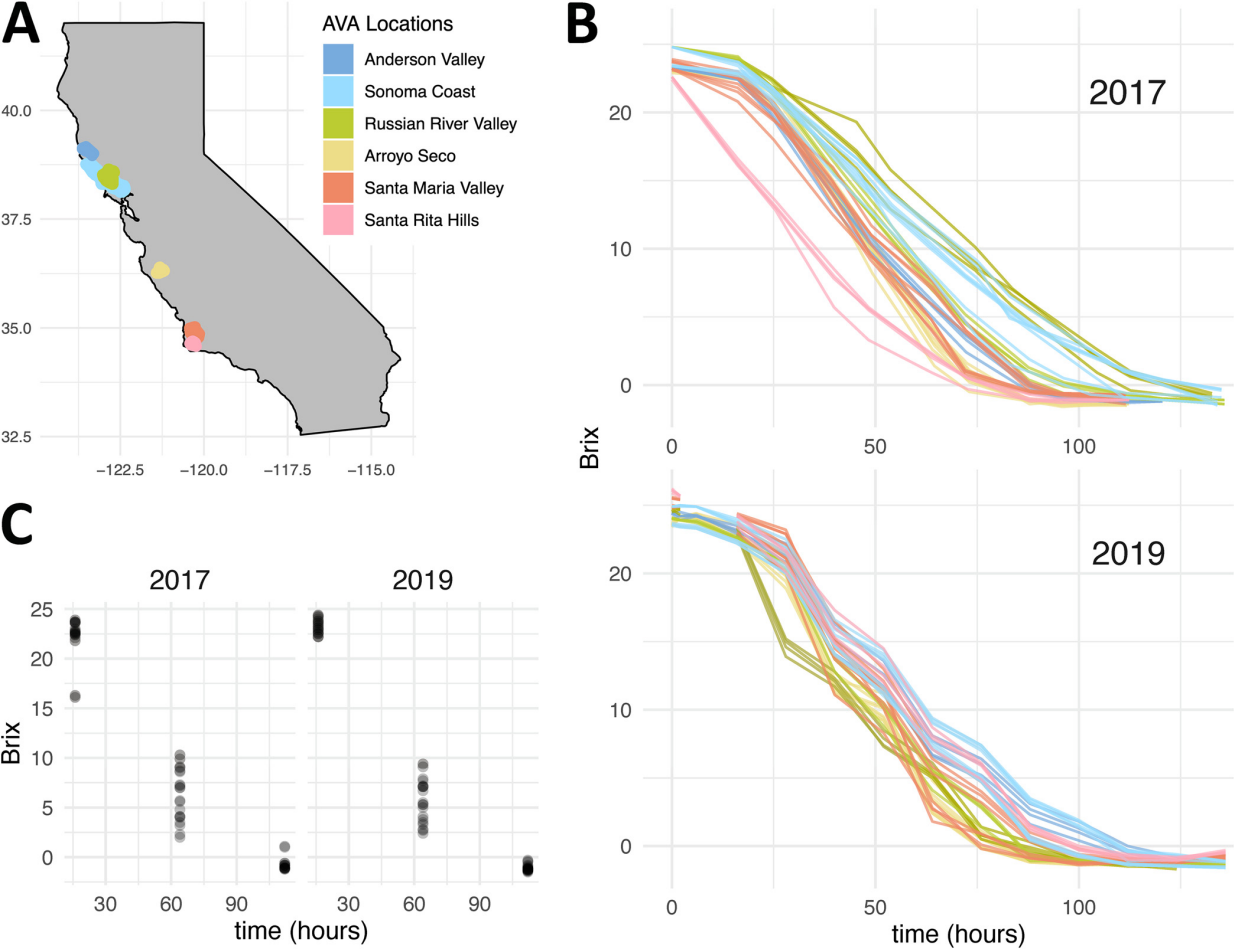
California vineyard locations and fermentation patterns. (A) Map displaying the six AVAs in which the 10 study vineyards are located. (B) Fermentation curves reflecting the change in Brix over fermentation. Brix is a measure of total soluble solids that is used as a proxy for sugar concentration in grapes, grape must, and wine. (C) Brix at the time of sampling for each RNA-seq sample, relative to inoculation. While samples were taken at the same absolute time, fermentations proceeded at different rates, leading to different Brix values in each fermentation.

Pinot noir grapes were harvested from the same 10 vineyards in California during the 2017 and 2019 vintages for wine production at the University of California, Davis, Teaching and Research Winery (Fig. 1A). To standardize fermentations, grapes from the same Pinot noir clone and rootstock were harvested at the same ripeness (∼24 Brix, using total soluble solids as a proxy for sugar concentration). Duplicate fermentations that used the grape material from each vineyard were sampled for a total of 40 fermentations (20 from each vintage) at industry-relevant scales using the same wine-making protocol. Each fermentation was inoculated with the commercial wine strain S. cerevisiae RC212 and sampled to collect cells for gene expression analysis (3′-biased transcriptome sequencing [3′-Tag-seq]) at 16 h (exponential phase/early fermentation), 64 h (stationary phase/mid-fermentation), and 112 h (decline phase/end of fermentation) postinoculation (Fig. 1B). While sampling times were standardized across fermentations, the rates of fermentation varied, resulting in samples being collected across a range of Brix values (Fig. 1C). Differences in fermentation rates likely reflect diversity in the starting material and different fermentation outcomes, which were also demonstrated in sensory studies performed on wines produced from these vineyard sites in previous vintages (50).

### Consistent whole-transcriptome remodeling occurs during fermentation, independent of vintage.

**FIG 2:**
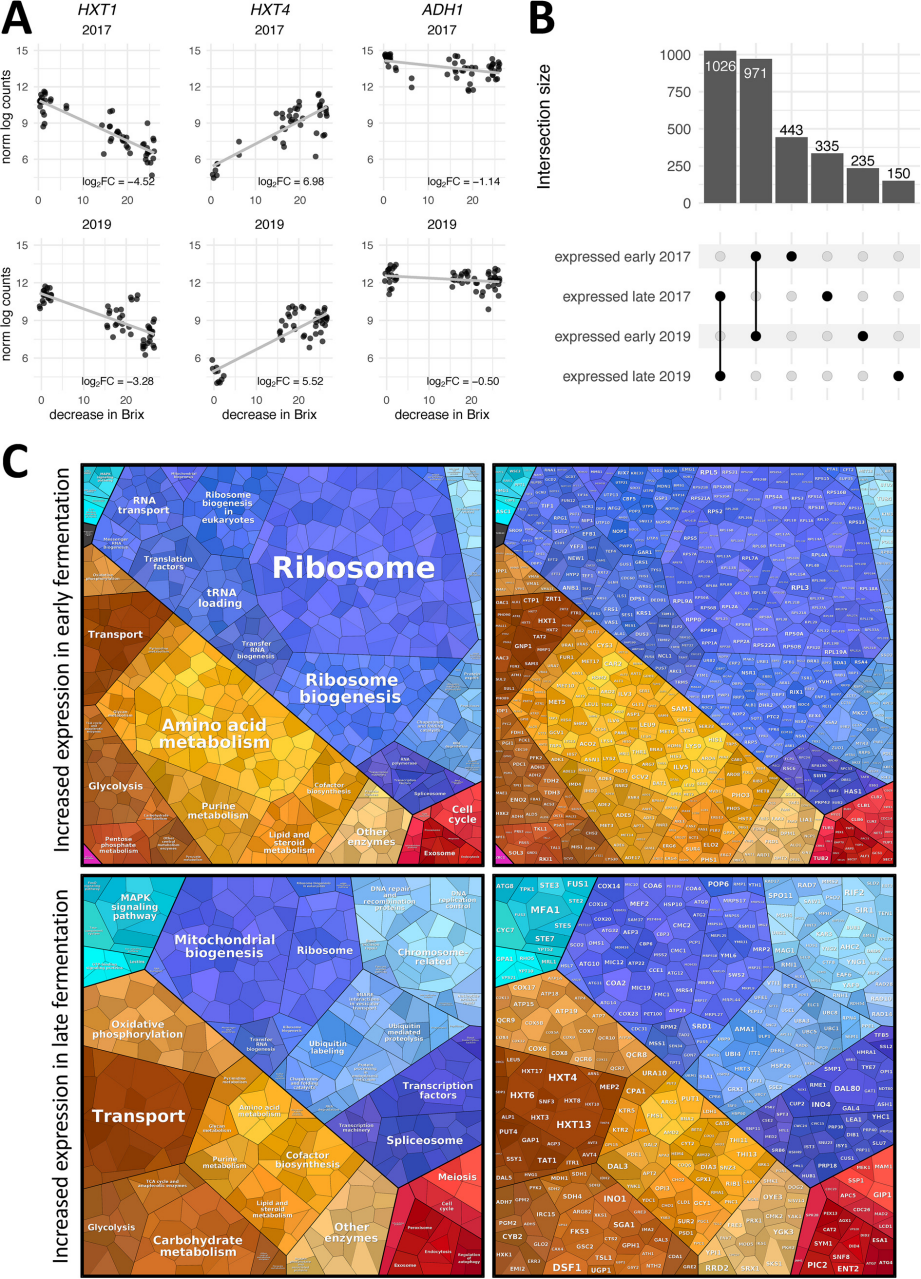
Transcriptome remodeling in fermentation is consistent across fermentations and vintages. (A) Graphs (*y* axis, normalized log gene expression counts; *x* axis, decrease in Brix over time) showing gene expression patterns in the 2017 and 2019 vintages, involving genes more highly expressed early (*HXT1*) and late (*HXT4*) in fermentation or constitutively (*ADH1*) across fermentation. (B) Upset plot showing the intersection of genes that are more highly expressed at the beginning and end of fermentation in each vintage, using a log_2_ fold change cutoff value of 1. The majority of genes are consistently expressed across fermentations and vintages. (C) Proteomaps depicting Gene Ontology pathways (left) and genes (right) that are more highly expressed in early (top) and late (bottom) fermentation. The sizes of individual genes reflect the associated log_2_ fold change values. Note that, for presentation purposes, not all genes that are significantly expressed are depicted. See Data Set S1 in the supplemental material for a complete list of genes.

The 3′-Tag-seq data from all 10 sites were combined and used to assess differential expression along the continuous variable Brix (Fig. 1C). This approach allowed incorporation and comparison of fermentations with disparate Brix values at mid-fermentation (Fig. 1B). These results define a core vineyard-independent gene expression program of RC212 during California Pinot noir fermentations. Under this model, log_2_ fold change values represent the changes in gene expression for each 1-unit decrease of Brix. Therefore, a positive log_2_ fold change corresponds to a gene that increased in expression as fermentation progressed, while a negative log_2_ fold change value corresponds to a gene that decreased in expression as fermentation progressed (see examples in Fig. 2A). After assessment of differential expression, the differentially expressed genes were intersected across vintages to determine consistent changes that were vintage independent. From this analysis, 971 genes decreased expression as Brix decreased, while 1,026 genes increased expression as fermentation progressed (Fig. 2B; also see Data Set S1 in the supplemental material). Each vintage also showed unique differential gene expression patterns, which may occur due to vintage-specific differences in fermentation. However, these data were generated at different times and newly developed methods were applied (unique molecular identifier [UMI] barcoding) (see Materials and Methods) for sequencing of the 2019 samples; therefore, it is suspected that the higher number of differentially expressed genes in the 2017 vintage may reflect differences in the quality of the sequencing data. Nonetheless, the large fraction of shared differentially expressed genes suggests that a core gene expression program is followed independent of vintage.

Of the genes that are differentially expressed in fermentation and shared across vintage, many are known to function in wine fermentation and are central to yeast growth, metabolism, and cell survival (Fig. 2B and C and Table 1). A strong signature of growth early in fermentation that included cellular investment in ribosome biogenesis, metabolism of lipids, purines, and amino acids, and cell division machinery was observed (Fig. 2C; also see Fig. S1 and S2). These processes, coupled with enrichment of associated pathways involved in RNA transcription and transport, reflect energy use for cell growth and proliferation associated with log-phase growth occurring in early fermentation. Further in fermentation, changes in ribosomal machinery gene expression occurred, as reported in previous studies (51) (Fig. 2C), reflecting a transition to a nonproliferative metabolic state. Late in fermentation, this was accompanied by changes in gene expression linked to nutrient limitation, altered metabolism, and entry into meiosis (Fig. 2C; also see Fig. S3 and S4), which included gene expression patterns consistent with hallmark isoform switches in hexose transporters and glycolytic enzymes that occur as concentrations of glucose or fructose change (52) (Fig. 2B and C). For example, *HXT1* encodes a low-affinity glucose transporter that was more strongly expressed at the beginning of fermentation, when glucose is abundant. HXT4 has a high affinity for glucose and is expressed when glucose concentrations are low (53), which was also observed in our data, as *HXT4* expression increased in late fermentation. Importantly, the pathways that were identified as enriched in early and late fermentation align with expectations based on previous research and the known biology of S. cerevisiae during fermentation (2–5, 13–16). This highlights the core processes that previous research efforts have defined and provides confidence that the analysis methods employed in these pilot-scale fermentations capture these biologically important transitions.

**TABLE 1:** Genes differentially expressed throughout fermentation shared across vintages

Cellular process and gene(s)	Expressed at highest levels	Gene product function
Constitutively expressed in fermentation
Glycolysis and fermentation
* HXT3*	Late	Hexose transporter induced by both high and low glucose concentrations.
* PFK1*, *PFK2*	*PFK2*, early	Phosphofructokinases that catalyze the first irreversible reaction specific to glycolysis, producing fructose-1,6-bisphosphate from fructose-6-phosphate.
* ADH1* to *ADH5*	*ADH2* to *ADH4,* early; *ADH5*, late	Alcohol dehydrogenase isoenzymes. The dominant fermentative alcohol is *ADH1* (138), responsible for reoxidation of NADH to NAD^+^, which is a required cofactor in the metabolism of glyceraldehyde-3-phosphate in glycolysis. *ADH2* is a nondominant isoenzyme of alcohol dehydrogenase. It is typically repressed by glucose and converts ethanol to acetaldehyde (138), and it is overexpressed in some wine strains (139).
Expressed at higher levels in early fermentation in both 2017 and 2019 vintages
Glycolysis and fermentation
* HXT1*	Low-affinity hexose transporter.
* HXK2*	Hexokinase that phosphorylates glucose in the first irreversible step leading to glycolysis.
* PFK1*, *PFK2*	Phosphofructokinases that catalyze the first irreversible reaction specific to glycolysis, producing fructose-1,6-bisphosphate from fructose-6-phosphate.
Acetate metabolism
* ALD4* to *ALD6*	Aldehyde dehydrogenase isoenzymes that produce acetate as a byproduct when acetaldehyde is metabolized. *ALD6* encodes the main isoenzyme responsible for acetate production in wine (98). Aldehyde dehydrogenase isoenzymes *ALD4* and *ALD5* are expressed when ethanol is the carbon source and are not typically associated with wine fermentation. Acetate contributes the majority of volatile acidity associated with negative organoleptic properties in wine (140).
* PDR12*	Plasma membrane ABC transporter that is required for development of resistance to weak organic acids, including acetate (141).
Cell cycle
* BAT1*	Expressed in logarithmic phase (142).
* CWP1*	Expressed in the S/G_2_ phase of the cell cycle (142).
Nitrogen metabolism
* GNP1*	High-affinity glutamine permease that also transports leucine, serine, threonine, cysteine, methionine, and asparagine.
* MUP1*	High-affinity methionine permease that is also involved in cysteine transport.
* CAR1*, *CAR2*	Involved in arginine catabolism. Arginine is the most abundant amino acid in grape must after proline (68) and is used in protein synthesis during fermentation (143).
* YPQ1*	Vacuolar transporter for arginine and lysine. Unused arginine is stored in the vacuole for later use (143).
Ehrlich pathway
* BAT1*, *ARO8*	Catalyzes transamination of amino acids, the product of which cannot be redirected to central carbon metabolism and so is excreted as fusel acid or fusel alcohol (144). Overexpression of *BAT1* increases the concentrations of isoamyl alcohol, its acetate ester, and isobutanol in wine (144).
* PDC1*	Catalyzes α-keto decarboxylation.
Glycerol biosynthesis
* GPP1*	Cleaves phosphate from glycerol-3-phosphate.
Expressed at higher levels in late fermentation in both 2017 and 2019 vintages
Nitrogen limitation
* GAT1*, *DAL80*	Transcriptional activator (*GAT1)* and repressor (*DAL80*) of genes under nitrogen catabolite repression. Expression is inversely correlated, and the detection of both genes as induced in late fermentation likely indicates tight transcriptional regulation of nitrogen metabolism.
* DAL2* to *DAL5*, *DAL80*, *DAL82*	Catalyze allantoin degradation, and expression is under nitrogen catabolite repression.
* MEP2*	Ammonia permease, and expression is under nitrogen catabolite repression.
* GAP1*	Amino acid permease.
* PTR2*	Peptide permease.
* AVT3*, *AVT4*	Vacuolar amino acid exporters that mobilize internal nitrogen stores for cell maintenance during stationary phase. Expression is under nitrogen catabolite repression.
Ubiquitin-mediated selective protein degradation
* RPN4*	Transcription factor that induces expression of proteasome genes.
* TMC1*	Effector of proteotoxic stress that is induced by nitrogen limitation, weak acids, and misfolded proteins and is a target of *RPN4*.
* UBC8*, *VID24*	Negative regulators of fructose-1,6-bisphosphate through ubiquitination (*UBC8*) and vacuolar targeting (*VID24*).
* UBC1*, *UBC5*, *UBC7*, *UBC13*	Ubiquitin-conjugating enzymes.
* UBI4*, *CUZ1*	Involved in the ubiquitin-proteasome pathway.
Autophagy
* ATG2*, *ATG4*, *ATG7* to *ATG12*, *ATG14*, *ATG32*, *ATG40*	Proteins involved in autophagy. Autophagy is a key response to nutritional limitation that allows cells to maintain homeostasis (145). Nitrogen starvation leads to the largest autophagic response in yeast.
Ehrlich pathway
* GRE2*	Final step of the pathway in which fusel aldehydes are oxidized or reduced to fusel acids or alcohols (144).
Carbon limitation
* SNF3*	Plasma membrane low-glucose sensor involved in regulating glucose transport.
* SKS1*	Serine/threonine kinase involved in the adaptation to low glucose levels via *SNF3*-independent signaling.
* PGM2*	Phosphoglucomutase that catalyzes a key step in hexose metabolism and is induced in response to glucose limitation and ethanol stress (146).
* HXK1*	Hexokinase that phosphorylates glucose or fructose in the first irreversible step leading to glycolysis, under glucose-induced repression.
* HXT4*, *HXT6*	Hexose transporters required at the end of alcoholic fermentation.
Trehalose and glycogen
* TSL1*, *NTH1*, *ATH1*	Involved in trehalose synthesis (*TSL1*) and degradation (*NTH1* and *ATH1*). Trehalose acts as a storage carbohydrate for cell maintenance under nongrowth conditions (147, 148), bolsters membrane integrity by displacing ethanol (148), and protects proteins from denaturation (149). Trehalose recycling is an important component of stress response (150).
* GLG1*, *GSY1*, *GSY2*, *IGD1*, *GPH1*, *SGA1*, *GAC1*, *GIP1*, *YPI1*, *PIG2*, *GLC8*	Involved in glycogen accumulation (*GLG1*, *GSY1*, *GSY2*, and *IGD1*), degradation (*GPH1* and *SGA1*), and metabolism (*GAC1*, *GIP1*, *YPI1*, *PIG2*, and *GLC8*). Glycogen accumulates during nutrient abundance and is metabolized during stationary phase and nutrient deprivation (147). Glycogen recycling is an important component of stress response (150).
Cell wall integrity
* PIR3*, *SED1*, *SLT2*	Target genes of the cell wall response to ethanol. *PIR3* is required for cell wall stability and is induced in part by *SLT2*. SED1 is a stress-induced cell wall structural protein (*SED1*) (151).
* PKH2*, *YPS1*, *PST1*, *KRE1*	General cell wall integrity response.

Beyond these previously defined core gene expression patterns, gene expression signatures indicative of less understood processes within these fermentations were also observed, which may be linked to the industry-like environment in which these studies were performed. These observations are discussed below.

### Nutrient limitation in early fermentation.

While gene expression data support logarithmic growth at 16 h postinoculation (Table 1; also see Fig. S1 and S2), at this early time point there is also evidence for the expression of genes that are typically upregulated in response to nutrient limitation. *PHO5* and *PHO89* encode phosphate transporters that are induced during phosphate starvation (54), both of which are expressed in early fermentation, along with *PHO90*. Phosphate limitation can cause stuck fermentations, because phosphate is critical for cellular function as a component of ATP, nucleotides, sugars, lipids, and macromolecules such as proteins (51, 55). Given that all of these Pinot noir fermentations went to completion and that the majority of glucose was converted to ethanol after the 16-h time point, induction of genes encoding phosphate transporters early in fermentation is not likely associated with phosphate starvation. Instead, it may be a response to the presence of non-*Saccharomyces* yeast, as cocultivation of S. cerevisiae with Torulaspora delbrueckii led to the induction of a gene encoding a high-affinity phosphate transporter (*PHO84*) after only 3 h of fermentation (42). Enological coculture of S. cerevisiae with organisms such as Hanseniaspora guilliermondii and *Brettanomyces* spp. has also been linked to induction of genes involved in vitamin biosynthesis in fermentation (56, 57), which could be indicative of increased nutrient competition and depletion of some nutrients early in fermentation. Induction of genes that encode enzymes involved in biosynthesis of B vitamins in early fermentation, including *BIO2* (biotin biosynthesis), *RIB3* and *RIB4* (riboflavin biosynthesis), *PAN6* (pantothenate synthesis), *SPE3* and *SPE4* (pantothenic acid synthesis), and *MIS1* and *FOL1* (folate biosynthesis) was observed. In addition, *THI21* was induced, which is involved in thiamine biosynthesis. As with phosphate, this may be related to the presence of metabolically active non-*Saccharomyces* microorganisms that were detected in all of these fermentations (58). We expect that continued work using industry-like fermentations across grape varieties and yeast strains, as well as controlled fermentations using reconstituted microbial consortiums, will be critical for understanding the relevance of these gene expression signatures to wine fermentation outcomes. If understood, such interactions could potentially be addressed through timely nutrient additions to a fermentation to achieve desired outcomes.

### Evidence of varied gene expression patterns linked to oxygen exposure during fermentation.

A wine fermentation is generally regarded as an anaerobic process, given that the carbon dioxide (CO_2_) produced as a by-product of ethanol fermentation protects must from dissolved oxygen (59). However, within anaerobia, there is an important distinction between hypoxic (low-oxygen) and anoxic (no-oxygen) conditions. In a fermentation, it is expected that molecular oxygen (O_2_) is introduced into the grape must by handling processes, including pump-overs, that may introduce small amounts of dissolved oxygen into industrial-scale tanks (60). Stratification within a fermentation may also expose local cell populations to different oxygen environments, leading to yeast cell populations undergoing different anaerobic processes. In this study, gene expression patterns were consistent with different populations of cells experiencing varied levels of oxygen exposure during fermentation. For example, the yeast cell wall undergoes remodeling in response to oxygen availability, which is accomplished in part by regulated expression of cell wall mannoproteins encoded by *CWP1*, *CWP2*, *DAN1*, and *TIR1* to *TIR4* (61). Specifically, expression of *DAN1* and *TIR1* to *TIR4* occurs reciprocally with expression of *CWP1* and *CWP2*, with the *CWP* genes being expressed under aerobic conditions and *DAN1* and *TIR1* to *TIR4* under anaerobic conditions (61). *DAN1* expression is known to be repressed under aerobic conditions by four independent regulatory mechanisms (62). Interestingly, expression of both *CWP1* and *DAN1* and *TIR1* to *TIR4* was observed in early fermentation samples. Similarly, in early fermentations, both *HYP2* and *ANB1* were expressed. These paralogous genes encode translation elongation factor eIF5A and are part of a family of paired genes for which oxygen induces the aerobic isoform and represses the hypoxic isoform (63). *HYP2* is expressed during aerobic growth, while *ANB1* is expressed during hypoxic growth and is tightly regulated by the presence of oxygen (64). Together, these gene expression patterns indicate varied gene expression programs within yeasts that may be explained by differing levels of oxygen exposure.

Among late expressed genes, oxygen-regulated paired isoforms, including *COX5A* and *COX5B*, which encode a subunit of cytochrome *c* oxidase, were also expressed. Modulated expression of these two isoforms allows S. cerevisiae to produce holoenzymes with different catalytic properties in response to oxygen (65). *COX5A* expression declines between 1 and 5 μmol/liter O_2_ and is undetectable below 0.25 μmol/liter O_2_, while *COX5B* is undetectable until 0.25 μmol/liter O_2_ (63). Simultaneous induction of both transcripts at the end of fermentation is again consistent with cells experiencing varied levels of dissolved oxygen in fermentation (60). In contrast, of the oxygen-regulated isoform pair *CYC1* and *CYC7* (63), only expression of the hypoxic isoform *CYC7* was detected at the end of fermentation. The breakpoint between expression of isoforms occurs at a higher concentration of 0.5 μmol/liter O_2_ for *CYC1* and *CYC7*, compared with *COX5A* and *COX5B* (63), which may indicate that dissolved oxygen levels did not exceed 0.5 μmol/liter and thus were not permissive for expression of *CYC1*.

In late fermentation, induction of pathways such as glycerol degradation and proline metabolism, which require oxygen, was also observed. Glycerol is a compatible solute involved in combating osmotic stress and redox balance and is primarily produced in early fermentation (66). *GCY1*, which encodes a glycerol dehydrogenase used under microaerobic conditions (67), was induced, as was *RSF2*, a transcriptional regulator of genes that encode proteins required for glycerol-based growth. Proline metabolism genes *PUT1*, *PUT2*, and *PUT4* were also expressed at the end of fermentation. Although proline is an abundant amino acid in grape must, it is a nonpreferred nitrogen source of yeast and requires oxygen to be metabolized (68). It was further observed that *PUT1* and *PUT2* were induced in a sealed laboratory wine fermentation but proline was not metabolized, given the absence of oxygen (2). Expression of *PUT1*, *PUT2*, and *PUT4* is regulated by nitrogen catabolite repression (69) and the presence of proline in the absence of other nitrogen sources (70) but is not regulated by the presence of oxygen. Intracellular proline accumulation also protects S. cerevisiae from reactive oxygen species (ROS) associated with ethanol-rich environments (71). While it possible that glycerol and proline were metabolized in late fermentation with oxygen ingress, other processes, such as nutrient limitation and oxidative stress, may also explain the induction of these genes.

Taken together, the gene expression data presented herein raise various questions about a distributed gradient of oxygen (hypoxia and anoxia) in the fermentation environment that may induce varied gene expression across the cell population. This could lead to yeast subpopulations with varied metabolic outputs or different levels of ethanol tolerance, due to the role of oxygen in these processes (72, 73). In the future, single-cell sequencing technologies combined with continuously monitored dissolved oxygen assays may help resolve these questions. From a production perspective, in industrial fermentations, even those that employ pump-over systems and thus maintain mixing and better homogeneity, there is a gradient of dissolved oxygen in the fermentation tank, with higher oxygen concentrations toward the top of the vessel (60). This suggests that heterogeneous gene expression profiles in response to oxygen would likely exist in these environments too. This is also an important fact to consider, because oxygen additions during fermentation are known to influence both fermentation and sensory outcomes. For example, in late fermentation, a single oxygen pulse increases the rate of fermentation mediated by ergosterol biosynthesis (72). Similarly, oxygen additions at different stages of fermentation differentially impact the formation of wine aroma compounds such as volatile thiols and esters; however, this appears to occur in a strain-dependent manner (73). This knowledge, combined with the impact of oxygen addition on fermentation outcomes, raises the idea that timely addition of oxygen may be a way to control fermentations rates and formation of wine aromas, which would be a tool easily accessible to winemakers.

### Mitochondria and fermentation.

In late fermentation, there was striking enrichment of pathways involved in mitochondrial biogenesis and function, as well as oxidative phosphorylation, among differentially expressed genes (Fig. 2C; also see Fig. S3 and S4). Substantial metabolic investment in mitochondrial systems suggests a critical role for mitochondria late in fermentation. What that role is remains unclear, however, as limited research has been conducted on the mitochondria during enological fermentation (74, 75). While some studies that profiled the transcriptomes of primary fermentations either found no evidence for or made no comment regarding enrichment for oxidative metabolism at the end of fermentation, many studies found induction of mitochondrial genes, particularly those encoding proteins involved in oxidative phosphorylation. These studies included fermentations conducted under nitrogen limitation (6), lipid limitation (76), and standard laboratory conditions (3). Interestingly, under lipid limitation, oxidative phosphorylation was induced in the exponential phase of growth, as opposed to the end of fermentation (76). Given the role of membrane lipid composition in combating ethanol-induced membrane permeability (77) and the accumulation of ROS during ethanol exposure (78), induction of the respiratory chain may mitigate ROS, which are abundant at the end of fermentation. Nonetheless, the recurrence of these gene expression patterns in our studies and previous laboratory experiments suggests that cells are investing in mitochondrial systems during fermentation.

One potential reason for late induction of mitochondrial systems is that glucose limitation relieves the Crabtree effect. This may lead to induction of oxidative phosphorylation genes that change metabolism in a nutrient-limited environment to one that generates the largest amount of ATP per unit of glucose (79). In this way, an investment in mitochondrial infrastructure during late fermentation may be a starvation adaptation in which S. cerevisiae uses oxidative phosphorylation to harness the largest fraction of energy possible from the remaining carbohydrate sources. However, this strategy is predicated on the availability of molecular oxygen, which is required for the induction and function of the respiratory apparatus (80, 81). A second reason for mitochondrial gene expression may be related to the fact that meiosis- and sporulation-related genes were enriched at the end of fermentation (Fig. 2C; also see Fig. S3 and S4). Induction of meiosis likely occurs to produce spores resistant to the challenges of nutrient limitation and stress (82). Interestingly, mitochondrial biomass is a predictor of meiosis (83), and components of the respiratory chain are required for initiation of sporulation (84), providing another potential process that may underlie mitochondrial investment in late fermentation. Related to this fact, a propensity for yeast to undergo meiosis at this stage of vinification underlies fast adaptive genomic evolution of S. cerevisiae (85), suggesting that this may be an important acquired trait that allows yeast to successfully survive the wine environment.

Mitochondria also fulfill other critical roles in fermentation that are unrelated to respiration. For example, mitochondria play a role in sterol uptake and transport under strictly anaerobic conditions (86), and mitochondria quench ROS, especially during ethanol stress (87). While we did not observe induction of specific genes related to sterol biology and we found induction of different genes related to ROS, compared to those identified previously (see below), these processes may also be linked to increased mitochondrial gene expression. Regardless of the role played by mitochondria in late fermentation, the striking and consistent induction of these genes in fermentations signals that more research is needed to understand the role of mitochondria in fermentation.

### Thioredoxin and glutathione system activity throughout fermentation.

The reducing environment of the cytosol in S. cerevisiae is key to various cellular functions, including deoxyribonucleoside triphosphate synthesis and the elimination of toxic compounds, including oxidants generated through cellular metabolism (88, 89). Key to maintaining redox balance are the thioredoxin (TRX) and glutathione (GSH) thiol reductase systems. For example, proper redox homeostasis is required to maintain the redox status of cysteine residues, which are essential for the function of numerous enzymes, protein receptors, and transcription factors. Similarly, redox homeostasis within cells aids to balance pools of reduced and oxidized pyridine nucleotide cofactors (NAD^+^/H and NADP^+^/H) that are essential to numerous metabolic reactions. ROS can alter this redox balance, causing oxidative stress and direct or indirect ROS-mediated damage of nucleic acids, proteins, and lipids. While typically associated with respiratory metabolism, ROS can be generated throughout fermentation, particularly by superoxide anions and peroxides (78, 90, 91). ROS may also be created by acetaldehyde, an intermediate in ethanol production (92).

In early fermentation, genes involved in the TRX system, such as *TRX1* and *TRR1*, were induced. Expressed targets of *TRX1* included *RNR1* to *RNR4* (93), genes encoding ribonucleotide diphosphate reductases required for DNA synthesis and cell cycle progression, as well as *MET16*, which encodes an enzyme required for sulfate assimilation (94). We further observed genes encoding Trx1 target peroxidases (*TSA1*) and peroxiredoxins (*AHP1*) that are constitutively expressed throughout fermentation along with superoxide dismutases (*SOD1* and *SOD2*). An additional source of ROS is peroxisomes, which may generate hydrogen peroxide in early fermentation via β-oxidation of fatty acids. *CTA1*, which encodes a peroxisomal catalase, and *ANT1*, which encodes a peroxisomal transporter involved in β-oxidation of fatty acids, were expressed in early fermentation. A major factor used to maintain redox balance is NADPH, which provides reducing potential for the TRX system. It has been shown that metabolic intermediates in glycolysis can be rerouted to the pentose phosphate pathway to generate NADPH in response to oxidative stress (95–97). In this study, the pentose phosphate pathway was enriched among genes expressed in early fermentation (Fig. 2C; also see Fig. S1 and S2), which includes *GND1*, encoding an enzyme that catalyzes NADPH regeneration and is required for the oxidative stress response. Other expressed genes that encode enzymes acting downstream of *GND1* in the pentose phosphate pathway included *RPE1*, *TLK1*, *TLK2*, and *TAL1*.

Central to the GSH thiol reductase system is GSH, an abundant tripeptide that is conserved throughout eukaryotic and prokaryotic cells, with a critical role in redox control, but its physiological role is both diverse and debated (97). Genes encoding enzymes involved in the degradation (*DUG1* and *DUG2*), import (*OPT1*), and biosynthesis (*GSH1* and *GSH2* in the 2017 vintage) of GSH were expressed in early fermentation. Additional generation of NADPH in early fermentation may be supported by the transformation of isocitrate to α-ketoglutarate via *IDP1* in the mitochondria and export via *YMH2*, as both genes were also expressed. Genes encoding aldehyde dehydrogenases (*ALD5* and *ALD6*) are similarly expressed in early fermentation, and both may regenerate NADPH through the transformation of acetaldehyde to acetate. ALD6 is the dominant isoenzyme responsible for acetate production in wine (98).

Genes involved in GSH-mediated ROS mitigation were also induced in late fermentation. For example, a gene encoding cytosolic glutaredoxin (*GRX1*) was expressed in late fermentation. Unlike glutaredoxins in other species (e.g., mammals), yeast glutaredoxins do not function as deglutathionylase enzymes (99). Instead, induction of *GRX1* increases resistance to hydroperoxides by catalytically reducing hydroperoxides through GSH conjugation and using the reducing power of NADPH (100). In addition, the cytosolic peroxidase GPX1 was expressed. GPX1 uses both GSH and TRX, in combination with NADPH, for reducing power (101). *GPX1* is known to be expressed with glucose and nitrogen starvation (102), which coincides with peak peroxide formation in yeast during wine fermentation (90). While the gene expression data in this study support a role for cytoplasmic GSH during late fermentation, genes encoding mitochondrial peroxidin (*PRX1*) and TRX (*TRX3*) were also expressed. Prx1 buffers the mitochondria from oxidative stress and is reductively protected by GSH, TRX reductase (Trr2), and Trx3 (103). Taken together, these results suggest that cytoplasmic and mitochondrial systems may be integral to combating increased oxidative stress at the end of fermentation.

GSH is also important for maintenance of cellular functions via other systems. For example, methylglyoxal, a reduced derivative of pyruvic acid, is a by-product of glycolysis that may account for up to 0.3% of glycolytic carbon flux in S. cerevisiae (104). GLO2, an enzyme that catalyzes methylglyoxal degradation in a GSH-dependent manner, was expressed in late fermentation, as were GSH-independent systems involved in the degradation of methylglyoxal (GRE2 and GRE3). Genes that encode proteins involved in GSH homeostasis were also expressed at the end of fermentation, including *GEX1*, which encodes a proton/GSH antiporter (105, 106). *GEX1* is known to be induced during oxidative stress (105) and modulates formation of the aromatic thiol 3-mercaptohexan-1-ol from its glutathionylated precursor in wines such as Sauvignon blanc (106). Conversely, induction of a gene that encodes an enzyme that cleaves GSH (*GCG1*) was observed and may be involved in apoptotic signaling via ROS accumulation (107).

Together, these gene expression patterns highlight how intertwined redox homeostasis is with almost all core metabolic processes in S. cerevisiae, as most pathways require oxidation or reduction by a pyridine nucleotide cofactor during at least one reaction. For example, NAD^+^/H and NADP^+^/H participate in 740 and 887 biochemical reactions through interactions with 433 and 462 enzymes, respectively (108). It is also well documented that experimental perturbation of both NAD^+^/H and NADP^+^/H leads to changes in aroma compounds in wine and other fermented beverages (109–112). The observations presented here, conserved across many Pinot noir fermentations, involving genes engaged in redox balance and mitigation of oxidative stress via thiol reductase systems offer further evidence for the importance of these systems. These findings provide motivation for future studies of these systems in the context of wine production, which would include control measures to aid cellular control of redox and to mitigate oxidative cellular stress.

### Stress-associated gene expression during fermentation.

**FIG 3:**
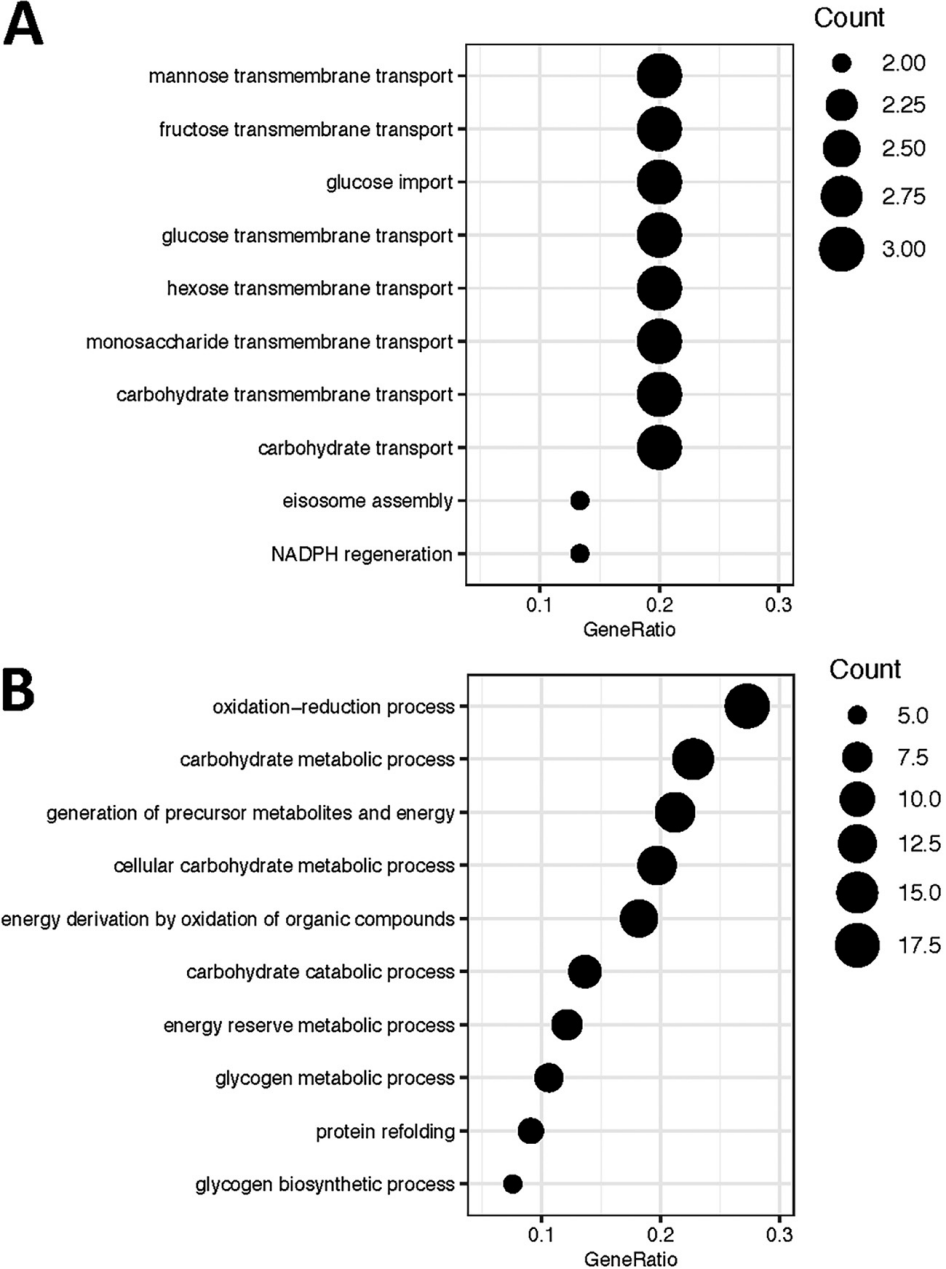
Pathways enriched among genes differentially expressed across fermentation that are shared with the ESR. (A) Of 16 genes that overlap the ESR and are expressed in early fermentation, pathways related to carbohydrate metabolism were enriched. (B) Of 78 genes that overlap the ESR and are expressed later in fermentation, pathways related to oxidation-reduction and carbohydrate metabolism were enriched. GeneRatio refers to the fraction of genes in an enriched gene set that were present in the tested set.

During fermentation, S. cerevisiae has to adapt to a continually changing stress landscape. Macronutrients and micronutrients become limiting as ethanol concentrations increase and, as discussed above, production of acetaldehyde and other metabolic processes generate oxidative stress. To accommodate this dynamic environment, S. cerevisiae wine strains express genes that overlap, but are distinct from, the stress response of laboratory strains (7, 113). In accordance with previous studies (2, 3), a partial overlap was observed between genes expressed in fermentation and those involved in the ESR in laboratory strains. Specifically, 16 ESR genes were expressed at the beginning of fermentation and 78 ESR genes were expressed at the end of fermentation. This matches observations in synthetic must, in which stress genes were induced upon entry into stationary phase (2). Stress-related genes expressed at the beginning of fermentation were enriched for Gene Ontology pathways involving carbohydrate (mannose, fructose, glucose, and hexose) transmembrane transport and NADP regeneration (Fig. 3A), while stress-related genes expressed at the end of fermentation were enriched for oxidation-reduction processes, generation of precursor metabolites and energy, energy reserve metabolic processes, and glycogen metabolic processes (Fig. 3B).

A recent study investigated the fermentation of Riesling grape must at laboratory scale without the addition of oxygen (3). Using microarray analysis at five time points in fermentation, the authors defined a fermentation stress response (FSR) as those genes that are induced at any point in fermentation and do not return to baseline (3). The FSR is differentiated from the ESR and the common stress response because adaptation over time through gene expression returning to prestress transcription levels is not observed (3, 7, 113). Of the 223 genes induced in the FSR, 84 were observed to be expressed in mid-fermentation or late fermentation. Of these 84 genes, 43 overlap genes expressed in other stress responses, as defined previously (3), including 16 with the ESR and 14 with the common stress response. Of the 41 genes that overlap the FSR, many were related to the challenging nutrient environment in wine, including glucose limitation (*NRG1*, *SKS1*, *HXT6*, and *VID24*), nitrogen limitation (*MEP2*, *GAP1*, *PTR2*, *AVT4*, and *VBA2*), vitamin limitation (*MCH5* and *VHR1*), and stress caused by heat, salt, protein misfolding, and cell wall defects (*GAC1*, *RPI1*, *JID1*, and *PSR2*). This suggests that multiple stress pathways are simultaneously activated by the challenging environment that S. cerevisiae encounters in wine fermentation, which likely defines the described FSR. Many genes identified in the FSR and expressed in this study during fermentation remain uncharacterized (*YPR152C*, *YBR085C-A*, *YDL024C*, *YDR042C*, *YMR244W*, and *YLL056C*), offering gene targets for future investigations related to adaptation to the fermentation and wine environment.

### Polyol metabolism in late fermentation.

**FIG 4:**
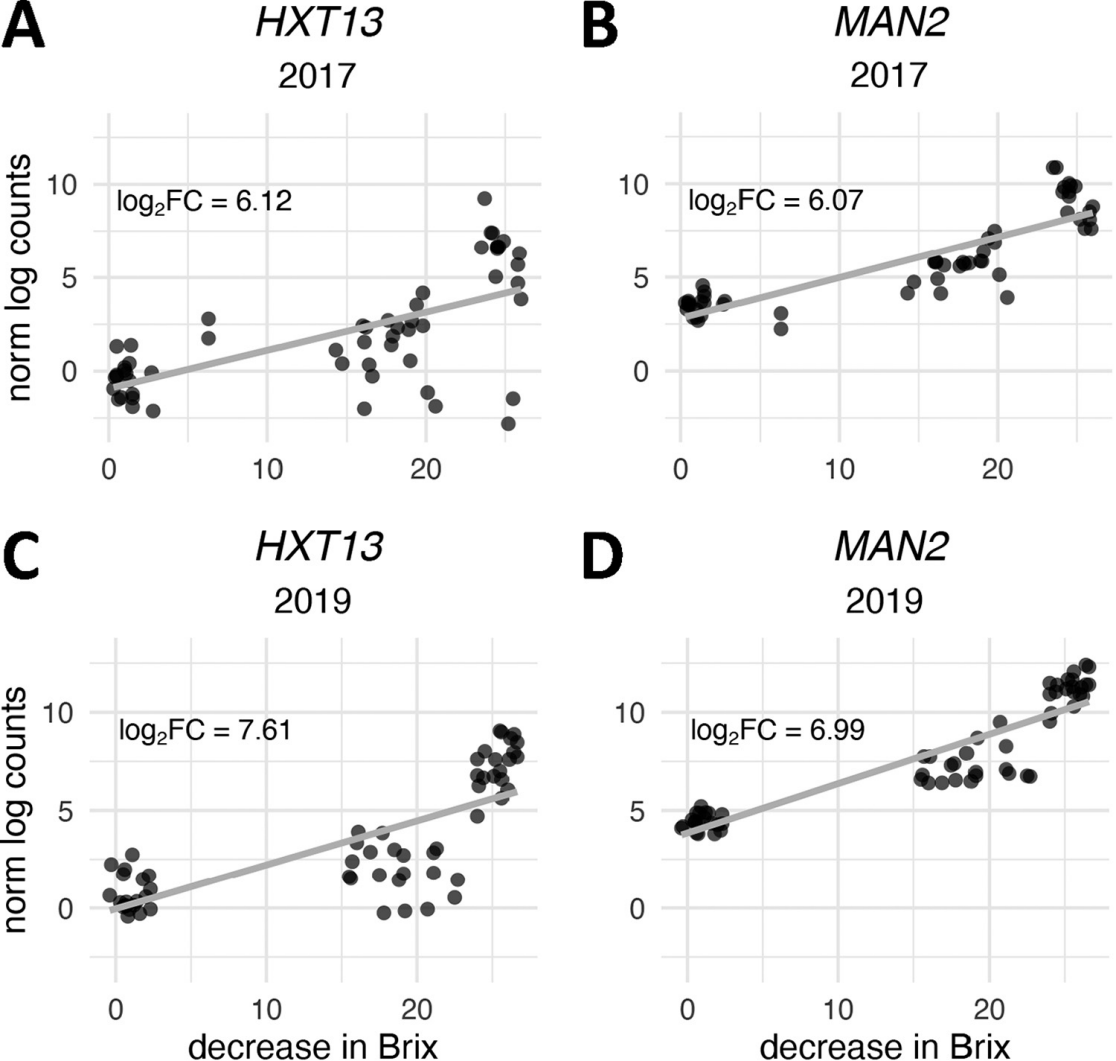
Normalized log gene expression counts for genes involved in mannitol transport and degradation. *HXT13* (A and C) and *MAN2* (B and D) expression in the 2017 (A and B) and 2019 (C and D) vintages is graphed. *MAN2* and *HXT13* were the most-expressed genes at the end of fermentation in 2019 and fell behind only *HXT4* in the 2017 vintage. Gray lines indicate a linear model fit to normalized counts.

Polyols, also called sugar alcohols, have recently been shown to be produced by non-*Saccharomyces* yeasts and by fructophilic lactic acid bacteria such as Lactobacillus kunkeei during fermentation (114, 115). Combined with other spoilage organism-associated metabolites, these compounds can have a significant impact on wine quality (116). Mannitol is one such polyol and a nonpreferred sugar that can be metabolized by S. cerevisiae (117–119). In S. cerevisiae, transporters encoded by *HXT13* and *HXT15* to *HXT17* were found to facilitate mannitol and sorbitol transport (118). In this study, the mannitol transporter gene *HXT13* was induced in both vintages, along with the mannitol dehydrogenase gene *MAN2*, which together indicate that mannitol may be present and metabolized by S. cerevisiae at the end of fermentation (Fig. 4). In line with this, although eukaryotic transcriptional profiling via 3′-Tag-seq was performed (see Materials and Methods), L. kunkeei transcripts were detected in some fermentations in both the 2017 and 2019 vintages (58), which is one potential source of mannitol production. These data raise the possibility of mannitol consumption by S. cerevisiae, demonstrating metabolic flexibility for carbon sources late in fermentation.

Notably, L. kunkeei can influence S. cerevisiae metabolism beyond the expression of genes for nonpreferred carbon sources. Via production of acetic acid and possibly other compounds, L. kunkeei has been shown to induce the [*GAR*^+^] prion phenotype in S. cerevisiae, thereby shifting carbon metabolism away from hexoses (120, 121). Given that the presence of L. kunkeei RNA was detected in the 2017 vintage, the presence of the [*GAR*^+^] phenotype was tested for in the 2019 vintage via cell culture (121). The [*GAR*^+^] prion was not detected in any fermentation tested in the 2019 vintage. While the absence of the [*GAR*^+^] phenotype in the 2019 vintage does not preclude its presence in the 2017 vintage, consistent gene expression for mannitol transport and degradation in both vintages suggests that S. cerevisiae may be metabolizing mannitol in these Pinot noir fermentations due to the presence of non-*Saccharomyces* organisms, including L. kunkeei.

### Vintage-specific differences.

From this data analysis, there were 778 genes and 385 genes differentially expressed in the 2017 and 2019 vintages, respectively. The majority of these genes were members of pathways enriched among all fermentations (Fig. 2C; also see Fig. S1 to S4). Using Gene Ontology enrichment analysis, no molecular function, cellular compartment, or biological process was enriched in either vintage that was not enriched in both vintages. This suggests that these differences may be largely due to sequencing depth or variations in the gene expression within these pathways and not differences in the overall biology of S. cerevisiae. Still, signatures indicative of vintage-specific effects, some of which may impact the sensory attributes of wine, were observed. For example, glycerol is an important fermentation by-product that can contribute to the mouth feel of wine (122). S. cerevisiae uses glycerol biosynthesis to generate NAD^+^, a required cofactor for glycolysis, when NAD^+^ levels are not sufficiently replenished through fermentation (123). During glycerol biosynthesis, enzymes encoded by *GPD1* and *GPD2* convert dihydroxyacetone phosphate into glycerol-3-phosphate (124). Both *GPD1* and *GPD2* were expressed in early fermentation in the 2017 vintage but not in the 2019 vintage. A second example involves genes encoding the fluoride transporters Fex1 and Fex2, which were expressed in late fermentation across all fermentations in the 2019 vintage. Fluoride is a toxic anion that S. cerevisiae exports via two plasma membrane transporters to avoid cell damage (125), which in excess can cause slow or stuck fermentation (126). Although fluoride is ubiquitous in terrestrial and aquatic environments (125), application of the insecticide Cryolite, which contains fluoride, has caused problematic fermentations in California vineyards (126). Currently, the reasons for these vintage-specific gene expression patterns are not known.

Finally, it was observed that genes of currently unknown function were differentially expressed in the two vintages assayed. Using a log_2_ fold change cutoff value of 2, 14 genes in the 2017 vintage and 7 genes in the 2019 vintage were of unknown function. Across both vintages, more genes of unknown function were expressed in late fermentation than in early fermentation (10 in 2017 and 5 in 2019). Knowledge of the specific pathways expressed in late fermentation due to the stressful, nutrient-limited conditions offers clues to the potential functions of these genes that could be explored in future work.

### Conclusion.

In this study, a gene expression analysis from 40 pilot-scale fermentations of California Pinot noir wine using grapes from 10 vineyard sites and two vintages is presented. The fermentations were diverse, with different kinetics, initial chemical conditions, and microbial communities (58). Yet among this diversity, a core gene expression program by S. cerevisiae that is largely consistent with that observed at laboratory scale was detected (2–4). Given that there were many genes consistently expressed across these Pinot noir fermentations from diverse vineyards, members of this core fermentation gene program represent strong candidates for future study to impact wine outcomes, e.g., through manipulating the redox balance (109–112). Excitingly, this includes a large number of genes with unknown function that, through investigation, may provide new insights into the biology of S. cerevisiae.

The largest deviations from benchtop fermentations are likely attributed to activities of non-*Saccharomyces* organisms, but more research is needed to understand these complex ecological interactions and their impact on fermentation. The gene expression signatures around oxygen presence and metabolic availability also warrant further research, particularly into the role of the mitochondria in late fermentation (3, 6, 76). While few vintage-specific differences were detected between fermentations, we expect that there are vineyard-site specific deviations from the consistent patterns of gene expression described here. Given the variability in fermentation kinetics with respect to time of sampling, new methods will likely be needed to resynchronize stages of fermentation to enable cross-vineyard comparisons (4). Future work is also needed to extend these observations to other grape varieties and S. cerevisiae wine strains, which will define both the shared and unique facets of the core gene expression program in S. cerevisiae linked to these variables. With such information, the impact of an industrial wine fermentation environment on S. cerevisiae gene expression can be addressed and approaches that can be used to manage commercial fermentation outcomes can be defined.

## MATERIALS AND METHODS

### Grape preparation and fermentation.

The wine-making protocol used in this study was described previously (25, 50). The grapes used in this study originated from 10 vineyards in six American Viticulture Areas (AVAs) in California. All grapes were Pinot noir clone 667 rootstock 101-14. Grapes were harvested at approximately 24 Brix, and the fruit was transported to the University of California, Davis, Teaching and Research Winery for fermentation. Separate fermentations were performed for grapes from each site, with two fermentations per site, totaling 20 fermentations per vintage (40 fermentations in total). After harvest, the fruit was separated into one-half-ton macrobins on harvest day, and Inodose SO_2_ (potassium metabisulfite and potassium bicarbonate) was added to achieve SO_2_ levels of 40 ppm. The bins were stored in a 14°C cold room until destemming and division of the fruit into temperature-jacket-controlled tanks. N_2_ sparging of the tank headspace was performed prior to fermentation, and the tanks were sealed with a rubber gasket. Grapes were cold soaked at 7°C for 3 days, with SO_2_ additions made on day 2 of the cold soak to maintain a level of 40 ppm total SO_2_. On the morning of day 3, musts were warmed for inoculation to 21°C with programmed pump-overs used to hold the tank at a constant temperature. Once the musts reached that temperature (∼2 to 3 h), the musts were inoculated. For inoculation, S. cerevisiae RC212 (Lallemand) was reconstituted with Superstart Rouge (Laffort) at 20 g/hl and the must was inoculated with 25 g/hl yeast. Superstart Rouge is a yeast preparate for active dry yeast rehydration. Fermentation progress was determined by measuring Brix with a density meter (Anton Paar 35 DMA). At approximately 24 h after inoculation, the nitrogen content in the fermentations was adjusted by adding diammonium phosphate (DAP), according to the formula (target yeast-assimilable nitrogen [YAN] −35 mg/liter – initial YAN)/2, and Nutristart (Laffort) using 25 g/hl. Nitrogen was adjusted only if the target YAN level was below 250 mg/liter based on measures of ammonia and free α-amino nitrogen content (Gallery automated photometric analyzer; Thermo Fisher Scientific). Approximately 48 h after fermentation, fermentation temperatures were permitted to increase to 27°C and DAP was added as described previously. Fermentations ran to completion when Brix was <0. Fermentations were sampled for Brix measurements and RNA isolation at 16, 64, and 112 h relative to inoculation. To ensure uniform sampling, a pump-over was performed 10 min prior to sampling of each tank. For RNA samples, 12 ml of juice was obtained and centrifuged at 4,000 rpm for 5 min. The supernatant was discarded, and the pellet was frozen in liquid nitrogen. Samples were stored at −80°C until RNA extraction.

### RNA extraction and sequencing.

Frozen yeast pellets were thawed on ice, resuspended in 5 ml Nanopure water, and centrifuged at 2,000 × *g* for 5 min, and the supernatant was aspirated. RNA was extracted using the Quick RNA fungal/bacterial miniprep kit, including DNase I column treatment (catalog number R2014; Zymo Research). Samples were eluted in 30 μl of molecular grade water and assessed for concentration and quality via a NanoDrop spectrophotometer and RNA gel electrophoresis. Sample concentrations were adjusted to 200 ng/μl, and 20 μl was sent for sequencing. 3′-Tag-seq (Lexogen QuantSeq) was used in both the 2017 and 2019 vintages, with the addition of UMI barcodes in 2019. The University of California, Davis DNA Technologies Core performed all library preparation and sequencing.

### Differential expression analysis.

Samples were processed according to manufacturer’s recommendations (Lexogen). First, the first 12 bp was hard trimmed from each read and Illumina TruSeq adapters and poly(A) tails were removed. Next, STAR was used to align the reads against the S. cerevisiae S288C genome (reference genome R64 [GenBank accession number GCF_000146045.2]) with parameters –outFilterType BySJout –outFilterMultimapNmax 20 –alignSJoverhangMin 8 –alignSJDBoverhangMin 1 –outFilterMismatchNmax 999 –outFilterMismatchNoverLmax 0.6 –alignIntronMin 20 –alignIntronMax 1000000 –alignMatesGapMax 1000000 –outSAMattributes NH HI NM MD –outSAMtype BAM SortedByCoordinate (127). For the 2019 vintage, UMI-tools was used to deduplicate alignments (128). Reads mapping to each open reading frame were quantified using htseq-count (129). Counts were imported into R and filtered to mRNA transcripts. To prepare for differential expression, the edgeR function calcNormFactors was used with default parameters (130). The limma package was used for differential expression by building a model using the decrease in Brix from initial Brix values; the data were prepared for linear modeling with the voom function, and a linear model was built for each gene with the lmFit function (131). Any gene with an adjusted *P* value of <0.05 was considered significant. To combat batch effects from different library preparation techniques used for the 2017 and 2019 vintages, differential expression was performed separately on counts from each vintage. The union of expressed and repressed genes was taken between vintages to generate the final set of differentially expressed genes. Expressed and repressed genes were visualized using proteomaps (132), and the intersection of differentially expressed genes between vintages was visualized using the R package ComplexUpset (https://github.com/krassowski/complex-upset). Gene set enrichment analysis was performed for genes that were expressed and repressed in both vintages against the Gene Ontology (ont = ALL) and Kyoto Encyclopedia of Genes and Genomes (organism = sce) databases using the R package clusterProfiler (133).

### Detection of Lactobacillus kunkeei in RNA-seq reads.

3′-Tag-seq sequences the tail end of transcripts that contain poly(A) tails. The majority of transcripts with poly(A) tails are eukaryotic in origin but, given that bacteria perform polyadenylation as a degradation signal (134), a very small subset of transcripts may originate from bacteria. Lactobacillus kunkeei was identified in RNA-seq reads using sourmash gather (135, 136). Using all L. kunkeei genomes available in GenBank (6 August 2019), sourmash signatures were generated for each using a k-mer size of 31 and a scaled value of 100. Sourmash index was then used to generate a database of L. kunkeei genomes, and this database was queried using signatures for each RNA-seq sample. To validate findings from sourmash gather, BWA-MEM was used with default parameters to map a subset of samples against the best-matching L. kunkeei genome (137).

### Culturing Saccharomyces cerevisiae for [*GAR*^+^] prion detection.

To ascertain whether the [*GAR*^+^] prion state was detectable in wine fermentations, yeast were cultured for the prion as performed previously (121). Yeast peptone-based medium containing the designated carbon source was used, such as YPD (1% yeast extract, 2% peptone, 2% agar, 2% glucose), YPG (1% yeast extract, 2% peptone, 2% agar, 2% glycerol), or GGM [1% yeast extract, 2% peptone, 2% agar, 2% glycerol, 0.05% d-(+)-glucosamine hydrochloride]. Yeast from fermentations were inoculated into each well of a 96-well plate containing 200 μl liquid YPD    plus 34 g/ml chloramphenicol, and then yeast were grown at 30°C for 48 h. Yeast were pinned to YPG or GGM plates and grown at 30°C for 4 days.

### AVA map construction.

The AVA map featured in Fig. 1 was constructed from the University of California, Davis, library AVA project (https://github.com/UCDavisLibrary/ava).

### Data availability.

RNA-seq data are available in the Sequence Read Archive (SRA) under accession number PRJNA680606. All analysis code is available at github.com/montpetitlab/Reiter_et_al_2020_GEacrossBrix.

## Supplementary Material

Download

Download
