# Supplementary material for: Saccharomyces cerevisiae Gene Expression during Fermentation of Pinot Noir Wines at an Industrially Relevant Scale
Source: Appl Environ Microbiol. 2021 May 11;87(11):e00036-21. doi: 10.1128/AEM.00036-21 (PMC8208162; doi:10.1128/AEM.00036-21)
Supplement: Download [file AEM.00036-21_aem.00036-21-s0001.pdf]

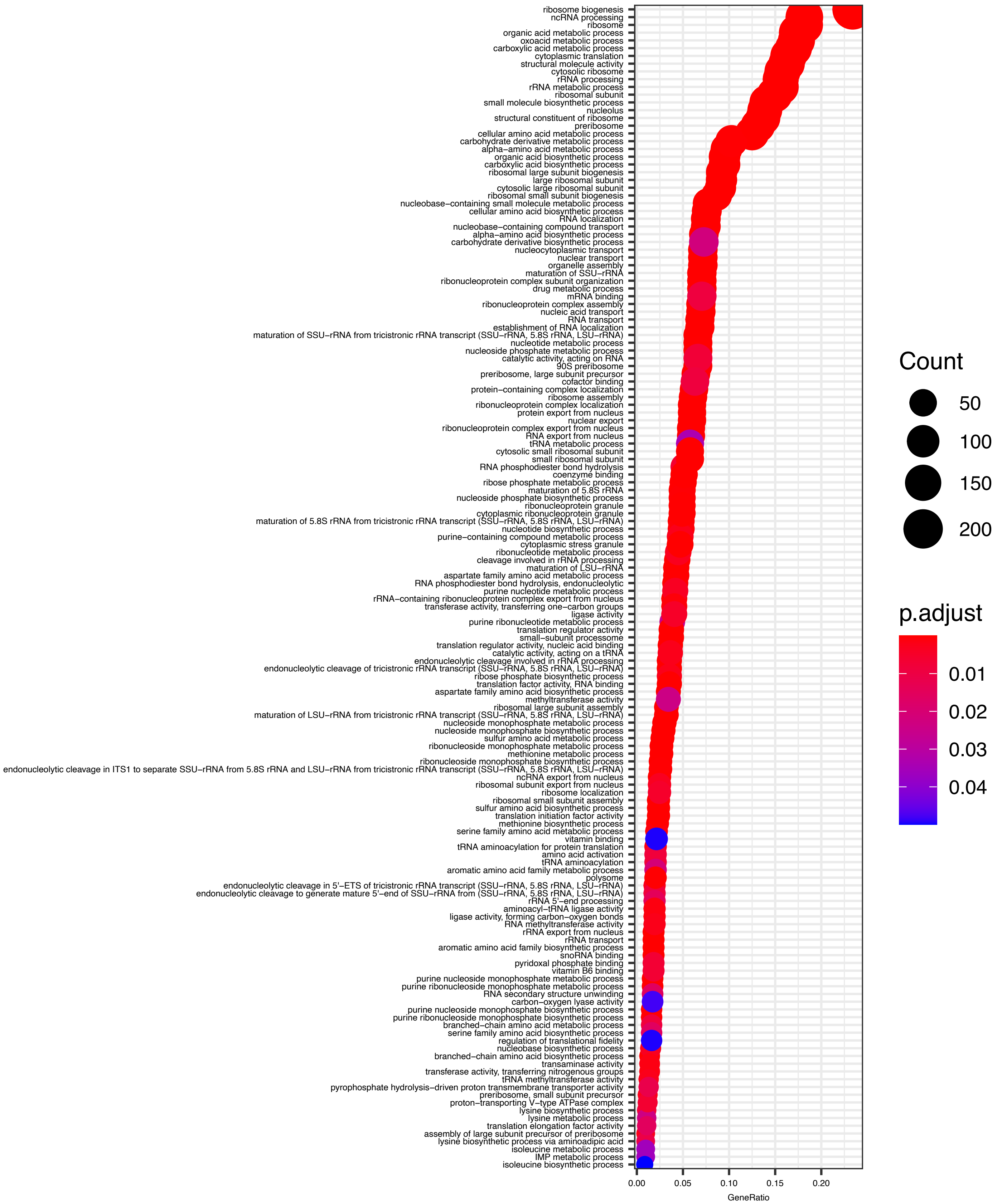

Figure S1. Significantly enriched Gene Ontology (GO) pathways in early fermentation. Pathways from GO categories molecular function, cellular component, and biological process are shown. Significant pathways are defined as $p < 0.05$ after Bonferroni p value correction. GeneRatio refers to the fraction of genes in an enriched gene set that were present in the tested set.

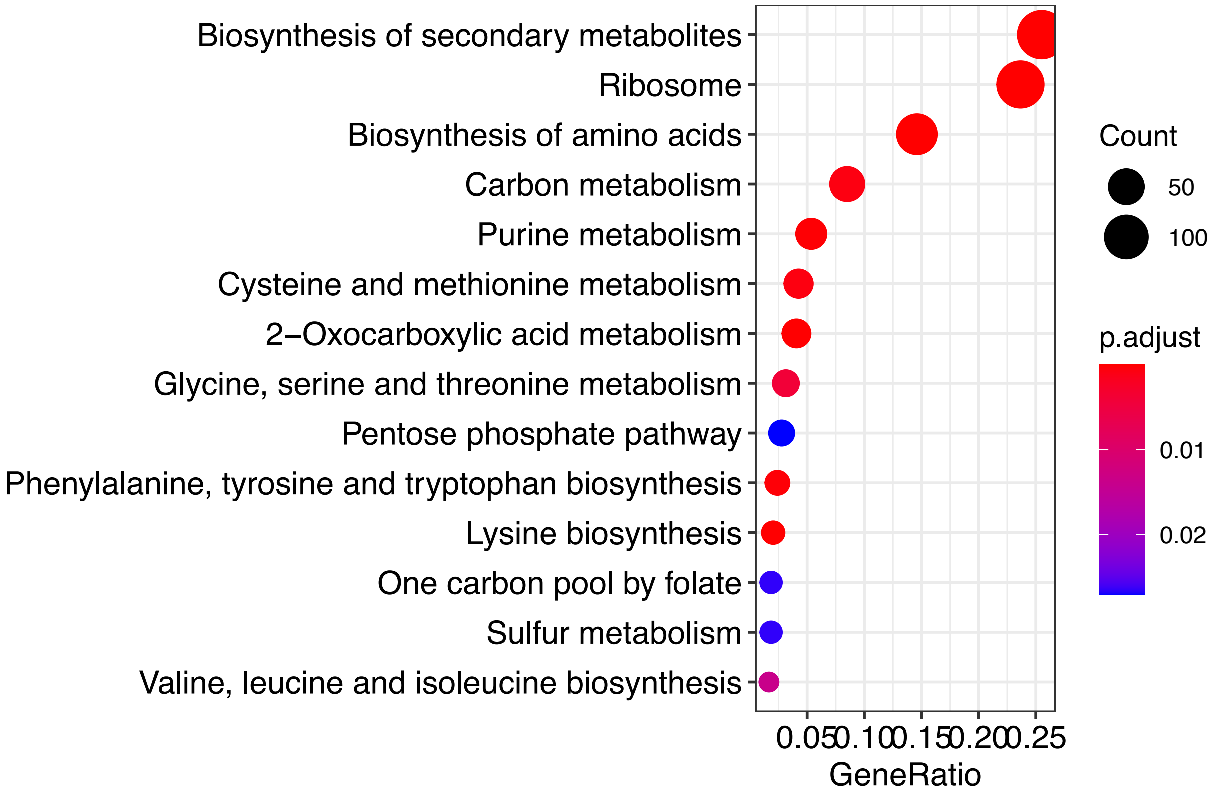

Figure S2. Significantly enriched KEGG pathways in early fermentation. Significant pathways are defined as $p < 0.05$ after Bonferroni p value correction. GeneRatio refers to the fraction of genes in an enriched gene set that were present in the tested set.

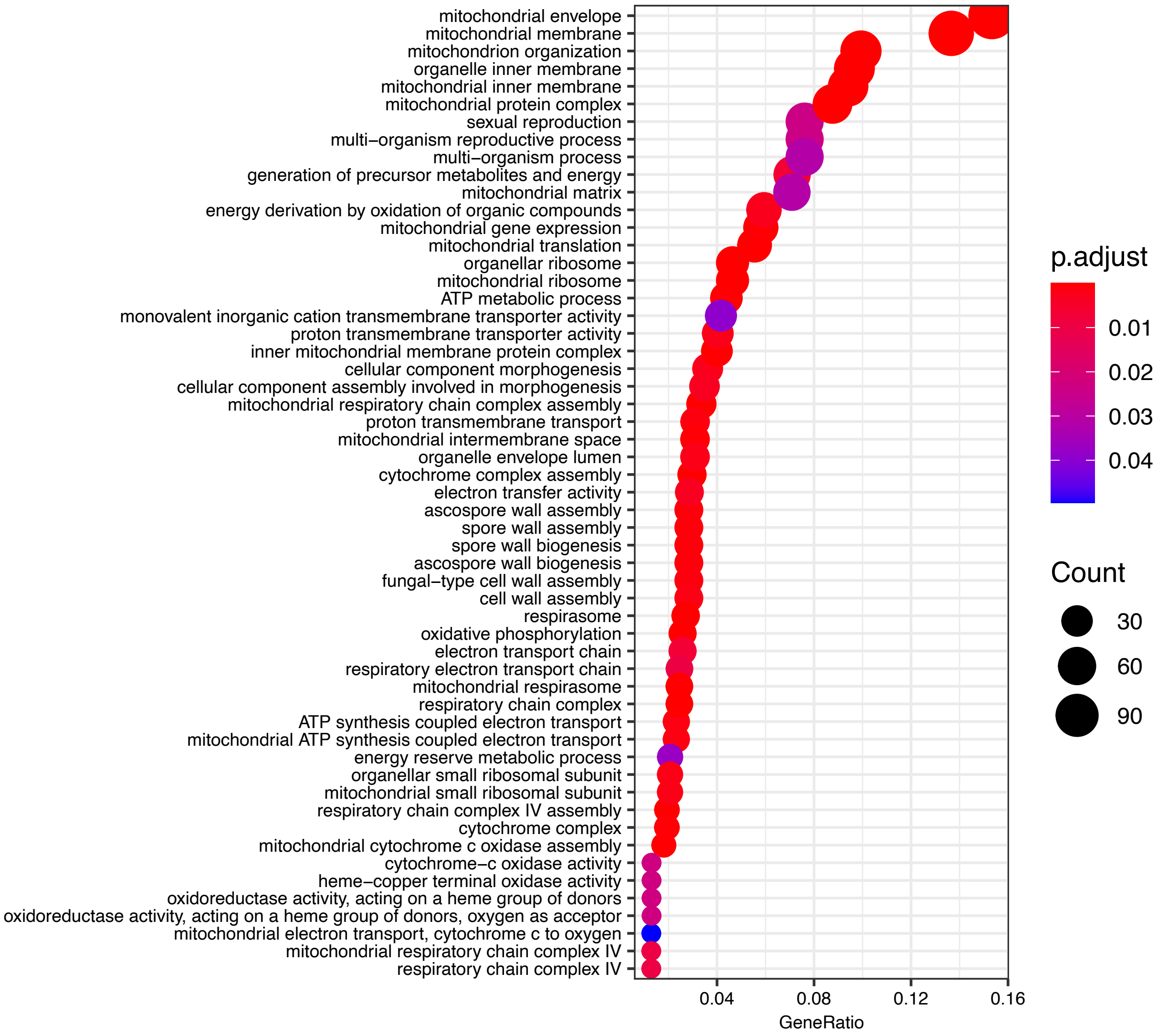

Figure S3. Significantly enriched Gene Ontology (GO) pathways in late fermentation. Pathways from GO categories molecular function, cellular component, and biological process are represented. Significant pathways are defined as $p < 0.05$ after Bonferroni p value correction. GeneRatio refers to the fraction of genes in an enriched gene set that were present in the tested set.

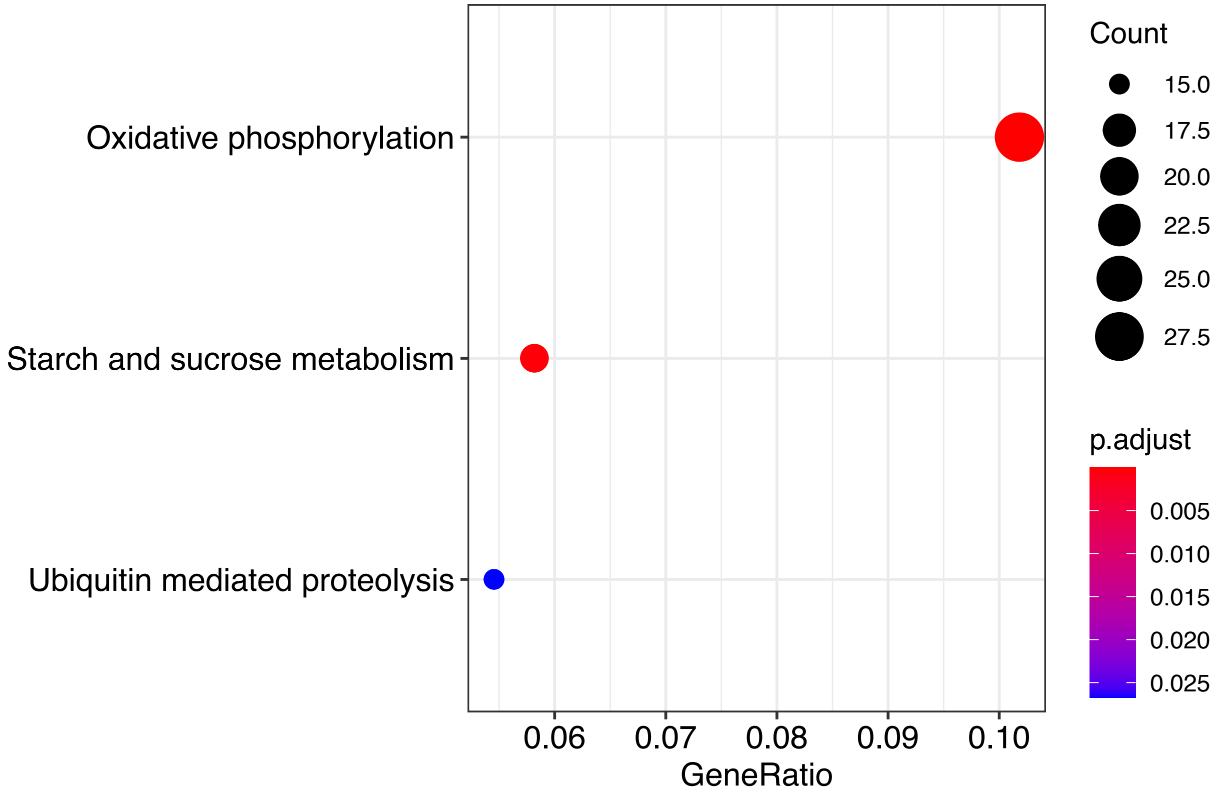

Figure S4. Significantly enriched KEGG pathways in late fermentation. Significant pathways are defined as $p < 0.05$ after Bonferroni p value correction. GeneRatio refers to the fraction of genes in an enriched gene set that were present in the tested set.
